# Raising Autistic Children in Mainland China: A Qualitative Study of Parental Experiences and Challenges

**DOI:** 10.1007/s10803-025-06877-x

**Published:** 2025-06-04

**Authors:** Yongshen Feng, Tsz Ying Christine NG, Dali LU, Lily Man Lee Chan, Junyan LI, Pui Hing Chau, Jojo Yan Yan Kwok

**Affiliations:** 1 School of Nursing, Li Ka Shing Faculty of Medicine, The University of Hong Kong, Hong Kong SAR, China; 2 Department of Child Mental Health, Shenzhen Longhua Maternity and Child Healthcare Hospital, Shenzhen City, Guangdong Province China; 3 Centre on Behavioral Health, Faculty of Social Sciences, The University of Hong Kong, Hong Kong SAR, China; 4 Department of Psychology, Xiamen Fifth Hospital, Xiamen, Fujian Province 361000 China

**Keywords:** Autism Spectrum Disorder, Parenting Experiences, Challenges, Qualitative Study

## Abstract

Purpose: Parenting children with autism presents unique challenges globally, particularly in China, where cultural factors and an underdeveloped service system can add complexity. This study aimed to explore the parenting experience and challenges of parents raising an autistic child in mainland China. Methods: A qualitative descriptive design using individual semi-structured interviews. Using purposive sampling, participants were recruited in mainland China from April to July 2023. Interview data were transcribed verbatim and analyzed using thematic analysis. Results: Twenty-five Chinese parents (nineteen mothers and six fathers) of autistic children were interviewed. Four themes emerged: (1) diminished quality of life due to the caregiving role; (2) fears and worries regarding children’s development; (3) experiences of isolation and loneliness in the caregiving journey; and (4) parental behaviors and their impact on child development. Conclusion: The findings suggest that effective support for individuals with autism extends beyond individual-level interventions, necessitating the integration of family care and robust community support systems. Cultivating autism-friendly communities – where understanding, acceptance, and resources are readily available – is crucial for a more inclusive and supportive environment that addresses cultural stigma, empowers caregivers, and ensures access to appropriate services.

## Introduction

Autism spectrum disorder (ASD) is a heterogeneous neurodevelopmental disorder typically characterized by restricted interests, repetitive behaviors, and deficits in social reciprocity and communication (Lord et al., 2018). The global prevalence of ASD is 1%, with a male-to-female ratio of 4.2 (Zeidan et al., 2022). Individuals with ASD often present with persistent comorbidities, including schizophrenia, language delay, depression, anxiety disorder, and attention deficits (Khachadourian et al., 2023; Yeh et al., 2023). Given these challenges, coupled with societal misconceptions and limited rehabilitation resources, daily caregiving for autistic children can be overwhelmingly demanding for parents (Marsack-Topolewski et al., 2021). Parents not only experience significant caregiving burdens but also face financial strains and psychological distress (Alnazly & Abojedi, 2019; Ou et al., 2015). Besides, parents may also have limited opportunities for leisure, difficulties adapting to family life, and strains in social relationships (Uysal et al., 2024). It is important to understand the specific health needs of parents of autistic children based on specific cultural contexts and guide effective interventions for those families.

### Parental Experience of Receiving Children’s ASD Diagnosis

Qualitative research on parental experiences following an ASD diagnosis has been conducted across various cultural contexts, though much of the literature originates from Western countries. For instance, a U.S. qualitative study that interviewed eight parents identified three key themes emerging from parental communication with healthcare providers: anguished questioning, urgently seeking help, and expecting a diagnosis (Johnson et al., 2020). A review of 36 qualitative studies highlighted barriers to obtaining an ASD diagnosis, including long waiting times, financial burdens, and insufficient specialized knowledge among healthcare providers (Smith-Young et al., 2024). Similarly, a review of 11 qualitative studies revealed that parents in the United Kingdom reported emotional, informational, and relational needs following their children‘s ASD diagnoses (Legg & Tickle, 2019). In Asian contexts, studies from Singapore and Hong Kong have provided valuable insights. A Singaporean study identified emotional, informational, and tangible needs among parents post-diagnosis (Goh et al., 2021), while research in Hong Kong highlighted challenges such as stigma and limited support systems (Ng & Ng, 2022). Despite these contributions, research on parental experiences and support systems during the diagnostic process in Asia, particularly in mainland China, remains limited, necessitating further exploration to address region-specific challenges.

### Psychological Burdens and Stigma Experienced by Parents of Autistic Children

Raising autistic children often leads to significant psychological challenges for parents. A meta-analysis of 31 studies revealed alarming median proportions of depression and anxiety among parents of autistic children of approximately 31% and 33%, respectively (Schnabel et al., 2020). Parents may experience emotional stages such as sadness, denial, shock, depression, and self-blame following their child’s ASD diagnosis (Uysal et al., 2024). The demands of daily caregiving for autistic children may contribute to heightened stress, sadness, frustration, and isolation (Uddin & Ashrafun, 2023). Besides, they often encounter stigma, which can further exacerbate their difficulties and negatively impact their mental and social well-being. A systematic review of 12 studies identified key stigma-related themes, including felt stigma, enacted stigma, variations in stigma, and contributions to stigmatizing experiences (Salleh et al., 2020). In Asian contexts, such as Hong Kong, stigma often stems from negative labeling by schools and healthcare professionals, peer bullying, and challenging behaviors of autistic children (Ng & Ng, 2022). However, the unique psychological challenges and stigma faced by parents in mainland China, where cultural attitudes toward developmental disorders differ, remain underexplored.

### Coping Strategies Used by Parents of Autistic Children

Faced with many psychological and life challenges, parents of autistic children employ various coping strategies. These strategies are often categorized as emotion-focused, which aims to manage emotional responses to stress (e.g., self-adjustment, acceptance, seeking emotional support), and problem-focused, which addresses the stressors directly (e.g., information seeking, skill development, advocacy) (Chin et al., 2023; Lin et al., 2008). A qualitative study in Singapore identified strategies such as adjusting psychologically, changing lifestyle to accommodate caregiving demands, actively contending with hurdles in accessing services, and seeking social support from family, friends, or professionals (Goh et al., 2021). Furthermore, research indicates that cultural values and socioeconomic factors can influence parenting styles and coping strategies. For instance, Chinese parents with higher social status and education tend to adopt authoritative parenting styles, potentially influenced by cultural values of collectivism and humility (Du & Li, 2023; Yim, 2022). Despite these insights, little is known about the specific coping strategies employed by parents in mainland China and their impact on family well-being.

### Research Gaps in the Current Qualitative Studies from Asia

While research has explored parental adaptation, unmet needs, emotional challenges, and coping strategies in families of autistic children, studies in Asia, particularly East Asian countries, remain limited compared to Western contexts. In Asia, parents face unique challenges, including high rehabilitation costs, limited access to medical and educational support, and cultural stigmas surrounding developmental disorders (Han et al., 2023; X. Li et al., 2024b; Ou et al., 2015). Within East Asia, studies from Singapore and Hong Kong have highlighted shared challenges, such as stigma and service access barriers, but mainland China presents distinct socioeconomic and cultural factors, including rural-urban disparities and traditional beliefs about disability (Li et al., 2024a; Liu & To, 2021). Li and colleagues interviewed 14 Chinese parents of autistic children aged 2–6 and identified five key caregiving needs: the acquisition of health- and disorder-related knowledge and information, maintenance of psychological and physical well-being, a desire for individualized caregiver skills training, the creation of multi-agent social support network, and the accessibility of high-quality and coordinated services (Li et al., 2024a). In contrast, another study highlights the potential for positive transformations following a child’s ASD diagnosis, including improvements in parental self-understanding, parent-child relationships, and a more profound life philosophy (Liu & To, 2021). However, comprehensive research on family support systems, psychological challenges, and coping strategies in mainland China remains scarce. Addressing this gap is essential to inform culturally sensitive, family-based interventions.

### Research Aim

Grounded in Ecological Systems Theory, this study posits that an individual’s development is influenced by interconnected environmental systems, ranging from the microsystem (e.g., self-coping, family support), to the exosystem (e.g., workplace, community service) and the macrosystem (e.g., society, culture) (Guy-Evans, 2024). This study aimed to explore parenting experiences and adaptation processes among parents raising autistic children in mainland China. By situating these experiences within the broader Asian context, the findings will guide researchers and healthcare providers in developing holistic, family-based interventions tailored to Chinese families.

## Methods

### Design

This study employed a qualitative approach, utilizing semi-structured individual interviews analyzed via thematic analysis (Braun & Clarke, 2006).

### Sample Size and Participants

Recognizing that a sample size of 16 to 24 participants is generally sufficient to achieve data saturation (Hennink et al., 2016), we tentatively aimed to recruit 20 participants, ensuring diversity in sociodemographic backgrounds (e.g., age and gender). Eventually, 25 participants were recruited via purposive sampling, guided by specific inclusion and exclusion criteria (Palinkas et al., 2015). Participants were recruited if they were (1) parents of at least one child (aged 0–18) who has already been diagnosed with ASD by board-certified pediatricians in the hospital with legal diagnostic qualifications, according to DSM-IV, DSM-V, or ICD-10 guidelines (Lord & Bishop, 2015; Wilson et al., 2013); (2) The Childhood Autism Rating Scale (CARS) scores > 30, and it was completed by parents and used to measure children’s autism symptom severity; (3) serve as primary caregivers to their autistic children, dedicating a minimum of 20 h per week for at least one year; and (4) able to read and communicate in Mandarin, the primary language in China, ensures that the interview data is more in-depth and reflective of the cultural context of mainland China. Parents were excluded if they had current severe psychiatric conditions, such as schizophrenia or mania.

### Study Setting and Recruitment

From April to July 2023, purposive sampling was used to recruit participants from a hospital in Shenzhen City, a major city in mainland China with a large and diverse population, including a substantial number of migrant workers from various regions (Zhang et al., 2011). Given the study’s focus on the experiences and potential challenges of Chinese families with autistic children within balanced social and cultural backgrounds, Shenzhen’s diverse socioeconomic landscape presented an appropriate setting for recruitment. Recruitment was facilitated through collaboration with outpatient clinics and inpatient wards, with pediatricians and rehabilitation therapists referring potentially eligible participants during admissions and therapeutic procedures. Additional recruitment strategies included disseminating information via social media and telecommunication platforms, supplemented by poster placements. Recruitment ceased upon reaching data saturation (Hennink et al., 2016).

To ensure confidentiality and ethical sensitivity, face-to-face screenings were conducted by a trained research assistant in a private hospital meeting room. During these sessions, the research assistant assessed eligibility, explained the study’s objectives and procedures and provided detailed information on data confidentiality, potential risks and benefits of participation, the unconditional right to withdraw, and the study’s ethical approval. Eligible participants were then invited to provide written informed consent before participating in a semi-structured interview, conducted individually to maintain privacy and encourage open responses.

### Data Collection

Upon referral, appointments were scheduled in a private meeting room at the hospital. The first author-verified eligibility criteria, explained the study procedures, and obtained written informed consent from eligible participants before commencing individual, semi-structured interviews. Data were collected using a semi-structured interview guide (Table 1) comprising open-ended questions designed to elicit detailed accounts of participants’ parenting experiences and challenges. The interview questions, developed through an extensive literature review (Cloete & Obaigwa, 2019; Han et al., 2021), and consultations with experienced frontline physicians specializing in psychological and educational services for families of autistic children, focused on: (1) participants’ perceptions of the ASD diagnosis; (2) their experiences raising an autistic child; and (3) their reactions and coping strategies for parenting and life challenges.

**Table 1:** Interview guide

No.	Questions
1	What were your feelings and responses when your child was diagnosed with ASD?
2	How do your families and friends react to your child’s ASD diagnosis?
3	How is your quality of life? Has it changed after receiving your child’s ASD diagnosis?
4	What’s the relationship between you, your child, your families, and your friends?
5	What’s your caregiving tasks?
6	What are your worries about your autistic child?
7	What symptoms or behaviors of your autistic child bother you the most?
8	What’s the influence of raising an autistic child on your physio-psycho-social health?
9	Have you experienced any difficulties (e.g., emotional problems) in raising an autistic child?
10	How did you handle these difficulties and emotional problems?
11	How is your ability and resilience when coping with these challenges?

Participants were initially asked about their feelings and responses upon their child’s ASD diagnosis. Subsequently, they were invited to describe their child’s diagnosis and subsequent adaptation process. Further questions explored family reactions, adaptation to the diagnosis, and the impact on family relationships. To gather data on the parenting experience, participants were asked about their caregiving tasks, life difficulties, and the influence of ASD on their physical, psychological, and social well-being. Finally, participants were asked to describe the coping strategies they had employed to manage parenting and life challenges.

All interviews were conducted at the parents’ convenience, in the absence of medical staff or family members, to minimize potential influence on their responses. Each interview lasted 45–60 min and was audio-recorded with the participant’s consent. The researcher also recorded observational notes, including verbal and non-verbal cues, to capture nuanced emotional expressions.

### Data Analysis

Qualitative data were managed using NVivo 12.0 software. Thematic analysis, as outlined by Braun and Clarke (2006), guided the data analysis process, involving iterative cycles of coding, subtheme and theme generation, and abstraction. Interviews were transcribed verbatim from audio recordings by two independent transcribers unaffiliated with the research team. The first and second authors, both registered nurses with five years of qualitative research experience and expertise in family health, independently reviewed the transcripts multiple times to generate initial codes, ensuring a rigorous and systematic coding process. These codes, derived inductively, captured common and unique ideas present in the data. Subsequently, recurring codes were identified and consolidated into key themes and subthemes through discussion. Finally, codes, subthemes, and themes were compared, grouped, and refined iteratively until consensus was achieved. Data analysis was conducted in the original Mandarin Chinese to preserve cultural and linguistic nuances (Kwok et al., 2020). Final themes and illustrative quotes were translated into English using the forward-backward translation method, adhering to established best practices to ensure accuracy and fidelity (Buono et al., 2023).

### Ethical Considerations

The study was conducted following the Declaration of Helsinki. Ethical approval was obtained from the Institutional Review Board of our university. To ensure voluntary participation, all participants were provided with comprehensive study information and provided written informed consent. Participants were informed of their right to withdraw from the study at any time without penalty. Anonymity and confidentiality were maintained by removing all identifying information from the data and assigning study code numbers. All data files were encrypted and password-protected, with access restricted to the research team members.

### Rigor

The study’s rigor was ensured through a multi-faceted approach, drawing upon established criteria for trustworthiness in qualitative research (Lincoln & Guba, 1985). Specifically, we focused on enhancing credibility, dependability, confirmability, and transferability. To enhance the credibility of the findings, investigator triangulation was employed (Brown et al., 2015). Two researchers independently reviewed transcripts to minimize bias and ensure that interpretations were grounded in the data. Regular meetings were held to discuss interpretations, resolve discrepancies, and refine the coding scheme. Furthermore, data accuracy was verified by two research team members who were not involved in the initial transcription process. Dependability was addressed through a detailed audit trail. This included maintaining thorough records of the research process, including interview transcripts, coding decisions, meeting notes, and analytical memos. Confirmability, which refers to the neutrality of the findings and the extent to which they are shaped by the participants rather than the researchers, was promoted through reflexivity. The researchers engaged in critical self-reflection throughout the research process, acknowledging their own biases and assumptions and considering how these might influence the interpretation of the data. Furthermore, direct quotations from participants were used extensively to support the findings, allowing readers to assess the confirmability of the interpretations. To facilitate transferability, a rich and detailed description of the participants, setting, and data collection and analysis procedures has been provided (Hastings, 2010).

## Results

### Characteristics of Participants

A total of 25 Chinese parents (19 mothers and 6 fathers) were interviewed regarding their experiences and challenges in raising an autistic child. The participants’ characteristics are summarized in Table 2. The parents had a mean age of 36.3 years (standard deviation [SD]: 4.6), ranging from 28.1 to 43.2 years. All parents were married, and their educational backgrounds varied, with 11 (44.0%) having obtained a bachelor’s degree or higher and 11 (44.0%) being employed part-time or full-time. All parents were married, and 16 parents (64.0%) had a per capita monthly household income exceeding 10,000 CNY. Among the 25 children diagnosed with autism, 19 (76.0%) were boys and 6 (24.0%) were girls. The children’s mean age was 5.6 years (SD = 1.3), ranging from 3.3 to 8.3 years. The time elapsed from ASD diagnosis to data collection averaged 21.7 months, with a range of 0 and 44 months. Of the 25 children, six had been diagnosed with ASD within the past year. The mean CARS symptom severity score was 35, ranging from 31 to 47. Among these children, 19 (76.0%) had mild to moderate autism symptom severity, while the remaining six (24.0%) had severe symptom severity.

**Table 2:** Sample characteristics (*n* = 25)

Participant code	Parents’ gender/ relationship to autistic child	Age (years)	Education	Employment status	Marital status	Per capita monthly household income (CNY)	Children’s gender	Children’s age (years)	First child/number of children prior	Time duration from ASD diagnosis to data collection (months)	Symptom severity scores (Childhood Autism Rating Scale)/level
P1	Male/Father	32.0	Bachelor or above	Homemaker	Married	10,000–15,000	Male	4.5	Yes	18.0	47/severe
P2	Male/Father	42.1	Middle/High school	Homemaker	Married	5000–10,000	Male	6.9	Yes	44.0	47/severe
P3	Female/Mother	35.1	Bachelor or above	Homemaker	Married	15,000–20,000	Male	5.1	Yes	33.0	31/mild-moderate
P4	Female/Mother	28.8	Middle/High school	Homemaker	Married	5000–10,000	Female	4.7	Yes	31.0	39/severe
P5	Male/Father	35.5	Bachelor or above	Full-time	Married	2000–5000	Male	6.4	Yes	19.0	31/mild-moderate
P6	Female/Mother	34.6	Middle/High school	Full-time	Married	10,000–15,000	Male	7.0	No/1	20.0	41/severe
P7	Male/Father	30.4	Middle/High school	Full-time	Married	5000–10,000	Male	3.4	Yes	0.0	38/severe
P8	Female/Mother	40.3	Bachelor or above	Homemaker	Married	≥ 20,000	Male	5.8	No/1	42.0	32/mild-moderate
P9	Male/Father	32.4	Middle/High school	Homemaker	Married	10,000–15,000	Female	6.2	Yes	36.0	31/mild-moderate
P10	Female/Mother	37.0	Bachelor or above	Part-time	Married	≥ 20,000	Male	3.3	Yes	8.0	31/mild-moderate
P11	Male/Father	31.3	Bachelor or above	Full-time	Married	5000–10,000	Male	4.8	Yes	36.0	36/mild-moderate
P12	Female/Mother	28.1	Middle/High school	Homemaker	Married	≤ 2000	Male	5.1	Yes	20.0	31/mild-moderate
P13	Female/Mother	37.2	Middle/High school	Homemaker	Married	2000–5000	Male	4.7	Yes	17.0	37/mild-moderate
P14	Female/Mother	40.3	Bachelor or above	Homemaker	Married	10,000–15,000	Male	4.2	Yes	9.0	31/mild-moderate
P15	Female/Mother	41.3	Middle/High school	Homemaker	Married	10,000–15,000	Male	4.8	No/2	5.0	33/mild-moderate
P16	Female/Mother	39.9	Middle/High school	Part-time	Married	5000–10,000	Male	7.4	No/1	0.0	31/mild-moderate
P17	Female/Mother	39.5	Bachelor or above	Full-time	Married	10,000–15,000	Male	4.6	Yes	19.0	31/mild-moderate
P18	Female/Mother	42.5	Bachelor or above	Full-time	Married	5000–10,000	Male	5.6	No/1	36.0	36/mild-moderate
P19	Female/Mother	38.8	Middle/High school	Homemaker	Married	≥ 20,000	Male	7.3	No/1	0.0	31/mild-moderate
P20	Female/Mother	43.2	Bachelor or above	Part-time	Married	10,000–15,000	Female	6.7	No/1	40.0	37/mild-moderate
P21	Female/Mother	31.8	Middle/High school	Homemaker	Married	10,000–15,000	Male	5.1	Yes	23.0	35/mild-moderate
P22	Female/Mother	34.0	Middle/High school	Full-time	Married	≥ 20,000	Female	5.1	Yes	21.0	38/severe
P23	Female/Mother	38.4	Middle/High school	Homemaker	Married	≥ 20,000	Female	8.3	No/1	14.0	35/mild-moderate
P24	Female/Mother	31.7	Middle/High school	Full-time	Married	10,000–15,000	Female	4.8	Yes	16.0	33/mild-moderate
P25	Female/Mother	40.8	Bachelor or above	Homemaker	Married	5000–10,000	Male	7.8	Yes	36.0	31/mild-moderate

### Thematic Analysis

The thematic analysis revealed four overarching themes that capture the core experiences of these parents: (1) diminished quality of life due to the caregiving role; (2) fears and worries regarding children’s development; (3) experiences of isolation and loneliness in the caregiving journey; and (4) parenting strategies in raising autistic children. These themes, supported by illustrative quotes from the interviews, are discussed in detail below (Table 3).

**Table 3:** Themes and subthemes of interviews

Theme	Subtheme
Diminished quality of life due to the caregiving role	Career sacrifices and transition to full-time caregiving
	Time constraints and neglect of personal needs
	Deteriorating health due to prolonged stress and depression
Fears and worries regarding children’s development	Concerns over children’s schooling and educational integration
	Apprehension about future independence and self-sufficiency
	Fear of societal discrimination and stigma
Experiences of isolation and loneliness in the caregiving journey	Lack of spousal support and teamwork
	Extended family conflicts over treatment plans
	Lack of fully understanding from friends
Parental behaviors and their impact on child development	Parents’ overwhelming reactions exacerbate children’s symptoms
	Empathy and emotional stability as effective parenting strategies
	Differential parental treatment among siblings

### Theme 1: Diminished Quality of Life Due To the Caregiving Role

A dominant theme emerging from the interviews was the marked reduction in parents’ quality of life directly attributable to their extensive caregiving responsibilities. Many parents described making substantial sacrifices, often forgoing career opportunities and personal pursuits to prioritize their children’s complex needs. This theme encompasses the multifaceted challenges of balancing caregiving with personal well-being, financial stability, and future aspirations.

### Career Sacrifices and Transition To Full-time Caregiving

Participants described a significant shift in their life priorities, with most of their time, energy, and financial resources now channeled toward their children. Some parents had to forgo their careers to assume the role of full-time caregivers, resulting in a significant shift from their original life plans and aspirations. They prioritize their child’s well-being over their career development.

*After receiving the diagnosis of children’s autism*,* it certainly impacted my life. The biggest impact was on planning my future. I didn’t return to work because I needed to care for him. (Participant 14)*

Another parent echoed this sentiment, stating, *“Now*,* most of my time was spent taking care of her. Seeing the child experience lots of autism symptoms*,* I had no choice but to quit my job.” (Participant 20)*.

### Time Constraints and Neglect of Personal Needs

Beyond career sacrifices, parents also described feeling constantly overwhelmed and depleted, lacking the time and resources necessary to attend to their own needs. The relentless demands of caregiving often left them feeling emotionally drained and physically exhausted.

*Taking care of the child was more exhausting than my previous job. Previously*,* I had my own time after work. Now*,* I’m required to constantly care for him around the clock*,* leaving no personal time for myself. (Participant 1)*

This sentiment was further reinforced by Participant 3, who noted, *“I’m not working now. I need to spend so much time taking care of him. I can’t change the situation. I barely have any time for myself. I don’t like my appearance now*,* it’s not good.”* The constant, 24/7 nature of caregiving was emphasized by Participant 21: *“I am with him 365 days a year*,* 24 hours a day. Whether he is sick or undergoing interventions at the hospital*,* I’m there for him. Indeed*,* a lot of time and energy is spent on him*,* so I don’t have any time for myself*,* and it’s overwhelming.”*

### Deteriorating Health Due To Prolonged Stress and Depression

The persistent stress and lack of self-care took a significant toll on parents’ physical and mental health, leading to a range of health problems. Participants described experiencing chronic sleep deprivation, depression, increased susceptibility to illness, weakened immune systems, frequent headaches, and chronic pain.

*My focus was mainly on the child. Since taking care of him*,* my health has deteriorated. I was more susceptible to illness*,* and I suffered from issues like kidney deficiency and headaches. (Participant 13)*

*For three consecutive days*,* I was crying incessantly*,* both day and night. Subsequently*,* I experienced a compromised immune response. I consulted a physician who suggested that my symptoms could be attributed to depression and insomnia and recommended hospitalization for treatment. (Participant 20)*

### Theme 2: Fears and Worries Regarding Children’s Development

Adding to the already significant burden of diminished quality of life, another prominent theme was the pervasive sense of fear and worry that parents felt regarding their children’s future development. These concerns extended to various aspects of their children’s lives, including their educational prospects, ability to live independently, and potential for social acceptance.

### Concerns Over Children’s Schooling and Educational Integration

As their children approached school age, parents expressed deep worries about their children’s ability to integrate into mainstream educational settings, with concerns ranging from acceptance by schools to the adaptability of the academic environment. Parents feared that their children’s problem behaviors may lead to exclusion or forced transfer to special schools, further impacting their social integration and prospects.

*My child is just over 7 years old*,* and I’m unsure whether he would be accepted into kindergarten or elementary school. I recently visited some schools to inquire. If the child is too disruptive*,* they might be transferred to a special school or have to take a break from studies. (Participant 6)*

Concerns about academic regression and school readiness were also voiced:

*We’ve noticed our child’s ability has regressed*,* and we’re realizing he might not be able to attend elementary school. That’s going to put a lot of pressure on him. We’re quite concerned about his ability to start elementary school. (Participant 17)*

#### Apprehension About Future Independence and Self-sufficiency

Parents articulated deep concerns over their children*’*s ability to acquire skills and self-care abilities when they grow up, which are essential for future employment and survival. The worries were further intensified by the consideration of their finite lifespans and the future care of their children once they were no longer able to provide support.

*I get anxious. I worry that if the interventions don’t work well*,* there will be concerns about how she’ll survive in the future. I mean*,* how will she manage to live independently once she grows up? (Participant 22)*

*Our goal is for our child to be able to live a normal life. I’m very worried that he won’t be able to live independently if he doesn’t learn anything. We won’t be able to take care of him forever. (Participant 25)*

#### Fear of Societal Discrimination and Stigma

Parents were reluctant to disclose their child*’*s autism diagnosis as they were afraid of societal prejudice. This reticence is rooted in the anticipation of gossip and the spread of misinformation, which can lead to social isolation and further marginalization.

*We haven’t told others about our child’s Asperger’s diagnosis. Since we do business at the market*,* if you tell one person*,* it’ll spread to everyone. We’re very worried about the gossip and rumors. (Participant 23)*

*I might not be able to tell my colleagues and friends that my child has autism right now. I don’t dare to talk about it openly. I worry about what the public might think. People might look at your child through colored lenses*,* and there could be a sense of discrimination. (Participant 24)*

### Theme 3: Experiences of Isolation and Loneliness in the Caregiving Journey

The challenges of raising a child with autism can also lead to feelings of isolation and loneliness, highlighting the social complexities inherent in this caregiving journey. This isolation often stemmed from a lack of support from spouses, family members, and friends, leaving parents feeling alone and overwhelmed in their responsibilities.

### Lack of Spousal Support and Teamwork

Participants frequently expressed feelings of isolation and insufficient support from their spouses following their child’s autism diagnosis. Participants reported that their partners refused to communicate or collaborate on autistic children’s treatment plans, leading to significant marital discord and emotional distress. Participant 03 detailed her experience of isolation and resistance from partners while parenting an autistic child:

*I often feel as though I bear everything alone*,* as if I’m walking this path by myself. When my husband first learned of our child’s autism diagnosis*,* he was very resistant and unwilling to take action. We argued for two to three months*,* as he consistently opposed me. I wanted us to communicate and collaboratively find a suitable treatment plan*,* but he refused to engage in any discussion.*

Some participants revealed they received inadequate emotional support and understanding from their spouses and felt undervalued and unappreciated for their efforts. Lack of recognition for their struggles and burned burdens exacerbated their feelings of being overwhelmed and unsupported. Participant 25 described her experience of being denied by her partner.

*My husband doesn’t understand me*,* and he’s not very comforting. He pointed out what I was doing wrong and thought I had a bad temper and couldn’t teach my child well. He can even give me the feeling of pouring cold water on my enthusiasm when I want to do something.*

### Extended Family Conflicts Over Treatment Plans

Participants expressed a notable generational gap in treatment approaches for autism. Grandparents preferred traditional methods or non-intervention over standard behavioral interventions as most of them often believed that children were normal and misdiagnosed with autism. These family disagreements not only might cause emotional distress but also potentially delay the initiation of crucial early intervention plans.

*There are differences in opinion on the child’s treatment plan between the elderly and us. The grandmother thinks that sending the child back to their hometown to attend kindergarten and consulting an old Chinese medicine practitioner is enough. There’s no need to insist on going to a big hospital for treatment. (Participant 01)*

*At the beginning of seeking medical help*,* the most troubling issue for me was the obstruction from my family. Grandma did not support the child*’*s intervention*,* and they insisted that the child was normal. (Participant 13)*

### Lack of Full Understanding from Friends

Participants found that friends often struggled to truly support and understand the unique parenting challenges of raising an autistic child. Lacking personal and professional knowledge about autism, friends often fail to recognize children’s autism symptoms, resulting in a lack of full empathy and understanding. This situation may make parents hesitant to discuss their struggles, as they may feel judged and unable to openly share their inner concerns with friends. Consequently, parents may feel disconnected from their friends due to a lack of meaningful and in-depth communication.

*I told my friends that my child had been diagnosed with autism*,* but they believed that there was nothing wrong with my child. They think I*’*m the one with the problem. It is difficult for me to talk to my friends when I feel stressed. After all*,* their children are so good*,* but it is difficult to talk about the ASD condition of our children. (Participant 3)*

The frustration and sense of isolation were palpable:

*My friends didn’t understand me at all. They said it was just that your child spoke a bit late*,* and maybe he had low intelligence*,* right? They told me to look on the bright side*,* that if the child can support himself*,* that was enough. Those friends didn’t have an autistic child in their family*,* they truly didn’t understand me. (Participant 15)*

### Theme 4: Parental Behaviors and their Impact on Child Development

When faced with children’s behavioral and emotional problems, most parents are easily irritated and adopt unhelpful disciplinary actions that can escalate the child’s distress. However, parents’ empathy and emotional stability can mitigate the child’s psychological distress and de-escalate conflicts. Additionally, the disproportionate attention given to autistic children can lead to sibling resentment.

### Parents’ Overwhelming Reactions Exacerbate Children’s Symptoms

Parents often describe feeling overwhelmed and emotionally affected by their children’s outbursts, leading to reactions such as yelling or physical discipline to control the situation. However, these responses intensify the child’s emotional state, resulting in increased distress, fear, and further behavioral escalation.

*When the child acts up*,* it bothers me. I might lose control and end up scolding or hitting him*,* which makes him even more upset*,* wronged*,* and stubborn. (Participant 17)*

*When I yell at her*,* sometimes she seems indifferent. But if I yell too harshly*,* she’ll start crying and screaming*,* and her behavior becomes more extreme. (Participant 22)*

#### Empathy and Emotional Stability as Effective Parenting Strategies

In contrast to reactive responses, parents identified empathy and emotional stability as effective strategies for de-escalating conflicts and supporting their autistic children’s emotional well-being. Autistic children are prone to lose their tempers when encountering activities they dislike, like hair washing, due to their limited ability to articulate their emotions and internal states, which can result in emotional outbursts and aggressive behaviors as a form of communication. By empathetically vocalizing the child’s feelings and thoughts, parents can help the child feel understood and emotionally supported, alleviating their psychological distress. One participant described this approach:

*Sometimes*,* he behaves quite rigidly. For instance*,* when he doesn’t want to wash his hair and his emotions flare up*,* he can’t express it with his language. But only use aggressive ways. At such times*,* I can help him articulate his thoughts. For example*,* I can ask him*,* “Did you not like it when mom washed your hair just now?” By describing his current feelings and narrating for him*,* he feels much better. (Participant 14)*

Furthermore, parents emphasized the importance of maintaining emotional stability when dealing with conflicts. A calm and composed attitude can expedite the calming process, whereas a stern or angry approach can exacerbate emotional issues:

*When the child is acting out*,* I just need to not be affected because if I am influenced*,* both of us end up feeling uncomfortable*,* especially since I would deal with him more sternly. He wouldn’t feel good about it. In contrast*,* if I can control myself and not get angry*,* the child’s mood stabilizes much faster. (Participant 14)*

### Differential Parental Treatment among Siblings

Parents often find themselves allocating an amount of time and attention to their autistic child, inadvertently neglecting the emotional and developmental needs of their other children. This imbalance can lead to feelings of resentment and a lack of connection between family members.

*I spend most of my time taking care of him*,* and I’ve been neglecting his sister. He had a bad relationship with her sister. (Participant 2)*

*I’m mostly looking after him. Grandma takes care of his sister more. So*,* my daughter and I don’t have a good relationship—sometimes we’re like enemies. (Participant 6)*

## Discussion

This study explored the experiences and challenges of parents raising an autistic child in mainland China. Upon receiving children’s ASD diagnosis, many parents choose to forgo their careers and personal time to ensure their children receive timely care and intervention. While caring for their autistic children, parents faced several challenges, including worries over children’s schooling problems and independent ability, fear of societal discrimination and stigma, and feeling isolated and lonely due to a lack of support systems. When dealing with their children’s emotional and behavioral problems, some parents find themselves easily irritated and resort to unhelpful parental behaviors like yelling and physical discipline. However, others discovered that maintaining empathy and emotional stability was effective in comforting autistic children. Furthermore, some parents realized that they inadvertently allocate unequal attention and affection to their autistic child and their siblings, leading to feelings of resentment among family members.

### Impact of Caregiving on Parental Well-being and Career

A central finding of this study is the significant impact of caregiving responsibilities on parents’ quality of life, with many adjusting or abandoning their careers and sacrificing personal time to care for their autistic child. This aligns with prior research in both Western and Chinese contexts (Gnanasekaran et al., 2016; Ozdemir & Koç, 2023; Zhao et al., 2024). In Western countries, policy supports such as paid and unpaid family leave, flexible work arrangements, and telecommuting help mitigate caregiving burdens, as noted in a U.S. study (Gnanasekaran et al., 2016). A Turkish study similarly highlighted the importance of career flexibility for parents facing heavy caregiving demands (Ozdemir & Koç, 2023). In contrast, Chinese parents in our study frequently reported complete career discontinuation due to the intense demands of caregiving. A Chinese cross-sectional study corroborated this, revealing high employment resignation rates with pronounced gender disparities: 54.3% of mothers (2,723/5,018) versus 2.8% of fathers (139/4,988) resigned due to caregiving responsibilities (Zhao et al., 2024). This phenomenon could be explained by that Chinese traditional values emphasizing family well-being and children’s education often lead parents, particularly mothers, to prioritize family obligations over personal career advancement and leisure (Han, 2012). The emphasis on early intervention before age six to optimize developmental outcomes (Maksimović et al., 2023) further drives parents to invest significant time in rehabilitation and education, often at the cost of psychological distress, including depression, due to overwhelming stress and self-neglect. Unlike Western systems with more robust workplace accommodations, the lack of such policies in China exacerbates these challenges, underscoring the need for tailored psychological support programs and flexible job skills training for full-time caregivers.

### Navigating the Educational Landscape and Societal Stigma

Parents in this study reported considerable concerns regarding their autistic children’s schooling challenges and future development, consistent with prior research (Attard & Booth, 2023; Ganjeh et al., 2020; Westby et al., 2024). They worried about their children’s future academic success, social integration, and independence, mirroring concerns in Western studies (Ganjeh et al., 2020). However, China’s educational landscape presents unique challenges. Parents preferred mainstream schools due to perceived inadequacies in the special education system, including insufficient policies, regulations, and qualified teachers (Xue, 2024). However, mainstream schools in China often lack specialized support, such as accessible teaching techniques, clearly defined educational responsibilities, and teachers trained in ASD-specific knowledge (Liu, 2023). These gaps hinder autistic children’s ability to adapt to school environments, exacerbating parental frustration and anxiety (Attard & Booth, 2023; Horgan et al., 2022). In contrast, Western countries like the U.S. and U.K. provide more robust educational support, including free integrated education and inclusive mainstream schooling (Horgan et al., 2022; Kim et al., 2019), while Asian countries, including China, South Korea, Taiwan, and Thailand, lag in inclusive education (Kim et al., 2019; Westby et al., 2024; Xue, 2024). These findings highlight the urgent need for China to strengthen inclusive education in mainstream schools and enhance resources in special education systems to better support autistic children’s academic and social development.

We found that the fear of societal discrimination and stigma are significant challenges for parents of autistic children, aligning with prior research (Ng & Ng, 2022; Salleh et al., 2020). Public awareness of autism is lower in China (57–65% of residents with sufficient knowledge) compared to the U.S. (86–91%) (Yu et al., 2020), with higher stigma levels (38% in China vs. 14% in the U.S.) (Lao et al., 2024). In China, ASD remains a highly stigmatized developmental disorder, manifesting as labeling, stereotyping, and social exclusion (Cleary et al., 2023; X. Li et al., 2024a, b). Consequently, parents often internalize the public’s negative attitudes, conceal their children’s ASD diagnosis, and experience affiliate stigma, resulting in reduced social engagement and self-esteem, and increased psychological distress (Turnock et al., 2022). Reducing this stigma requires widespread public awareness education to foster greater understanding and acceptance of ASD, thereby creating a more supportive and inclusive environment for autistic children and their families.

### The Critical Role of Social Support and Family Dynamics

We found that parents felt isolated and lonely due to insufficient support from spouses, friends, and society, consistent with previous research (Goh et al., 2021; Li et al., 2024a). The unique challenges of ASD, such as managing problem behaviors and navigating complex healthcare systems, can make it difficult for partners and friends to understand and provide the necessary support. Additionally, conflicts with elderly family members over treatment plans were common, with elders favoring traditional Chinese medicines (Feng et al., 2022) over evidence-based behavioral interventions advocated by parents and professionals (Leaf et al., 2022). These conflicts, exacerbated by the emotional and financial burdens of ASD care (Ou et al., 2015), contrast with Western systems, which offer more comprehensive social support, including free early interventions, individualized education programs, multidisciplinary teams, and peer support activities (Centers for Disease Control and Prevention (CDC), 2024). Strengthening China’s emotional and practical support networks through educational programs, family workshops, and parent-to-parent support, which a review of 25 studies found valuable for emotional support and advocacy (Lee et al., 2024), is essential to alleviate isolation and promote collaborative decision-making.

### Parental Behaviors: From Ineffective Reactions To Empathetic Approaches

Parents employed diverse parental behaviors to manage the emotional and behavioral challenges of autistic children. Some resorted to ineffective methods like yelling and hitting, while others adopted empathetic and emotionally stable approaches, aligning with studies advocating authoritative parenting and emotion-focused coping (Chin et al., 2023; Yim, 2022). The stress of raising an autistic child can prompt impulsive reactions (Miranda et al., 2019), particularly in China, where traditional norms view physical discipline as acceptable (Liu & Wang, 2018). In contrast, Western parenting literature emphasizes empathy and emotional regulation (Heiman, 2021; Yim, 2022), which our findings suggest enables parents to better meet their child’s needs, reduce stress, and foster healthier parent-child relationships (Lin & Faldowski, 2023). These findings underscore the need to provide psychoeducation and support to Chinese parents to develop empathetic parenting strategies to improve outcomes for both parents and children.

### The Impact of Differential Treatment on Family Dynamics

Another notable finding is the differential parental treatment of children, often prioritizing the needs of their autistic child over those of their typically developing children. This may be because parents’ attention was easily attracted by the need to address the specific challenges of autism, such as difficulties in communication and social interaction (Lord et al., 2018). Additionally, societal stigmatization of ASD in Chinese culture can heighten parental guilt and responsibility, further directing attention toward the autistic child (Ng & Ng, 2022). However, over-attention of autistic children can hinder their self-independence skills, while neglecting typically developing siblings can lead to resentment and strained family relationships (Rivers & Stoneman, 2008). Therefore, providing comprehensive support is crucial to promote healthier family dynamics and enhance the well-being of both children with ASD and their siblings.

### Impact of Family Factors on Parenting Experience

We found that 44.0% of parents had a bachelor’s degree or higher, slightly slower than the 54.0% reported in one Chinese study (Yan et al., 2023) and 69.7% in a US study (Marsack-Topolewski et al., 2021), but higher than the 33.33% in a Hong Kong qualitative study (Ng & Ng, 2022). The Chinese educational system and cultural emphasis on academic achievement place high expectations on parents for their autistic children’s educational performance, contributing to significant parenting challenges and stress (X. Li et al., 2024b). Furthermore, 64.0% of parents reported a per capita monthly household income exceeding 10,000 CNY (~ US$1,360, as of April 16, 2025), which is higher than the average of 5,130 CNY (~ US$698) reported in a study of 459 Chinese families with autistic children (Ou et al., 2015). However, this is considerably lower than the 59.1% of U.S. caregivers of autistic children reporting a monthly family income greater than 60,000 CNY (~ US$8,165) (Marsack-Topolewski et al., 2021). The relatively higher income among our participants may be attributed to their recruitment from a hospital in Shenzhen City, one of China’s top five cities by Gross Domestic Product (China Daily, 2023).

### Impact of Children Factors on Parenting Experience

In our study, among the 25 children, 19 had mild to moderate autism symptoms, and six had severe symptoms. Children with severe symptoms often require more intensive support, leading to increased parenting challenges, heightened caregiver strain, and the need for more robust coping strategies (Brunt et al., 2024). Families of children with severe symptoms (informants 1, 2, 4, 6, and 22) reported conflicts over treatment plans, limited caregiver self-care, poor school performance, problematic behaviors, and sibling conflicts, challenges less prevalent in Western contexts with stronger support systems. Additionally, six children were diagnosed within the past year, and their parents faced profound emotional challenges, including sadness and shock (Uysal et al., 2024), and significant emotional, informational, and relational needs (Legg & Tickle, 2019). For instance, informants 10, 14, and 15 left jobs to provide full-time care and faced social misunderstanding, while informants 7 and 16 reported emotional volatility. These findings, universal across cultures, are amplified in China due to limited post-diagnostic support, emphasizing the need for community and psychological services.

### Strengths and Limitations

This study had several strengths. First, this is the first to explore family support systems, psychological challenges, and coping strategies in families of autistic children within the Chinese context, contributing to the development of targeted family-based interventions. Second, participants were recruited from a hospital in Shenzhen City, a major urban center with a large and diverse population, including many residents who have immigrated from various provinces in China (Zhang et al., 2011). So, the sample is relatively representative of the diversity of Chinese societal culture. Finally, we selected fathers or mothers who are the primary caregivers of autistic children, which adds depth and richness to the findings.

However, this study also has several limitations. First, our participants were primarily recruited from a single hospital in Shenzhen, an urban city with greater service availability than in rural regions. This may limit the generalizability of the findings to families residing in rural areas of China. Future studies could recruit participants from multiple centers across China to enhance representativeness. Second, our sample included six fathers and nineteen mothers. Future research could consider recruiting fathers and mothers in an equal ratio or focusing exclusively on either fathers or mothers to obtain deeper insights from their perspective. Third, the study explored parental experiences with a time range from ASD diagnosis to data collection spanning 0 to 44 months, providing limited insight into the experiences specific to the first-year post-diagnosis. This broad range may influence the diversity of parenting experiences reported, as parents at different stages of adaptation may face distinct challenges, potentially affecting the generalizability of the findings. Future research should focus on the early post-diagnostic period to better inform timely and targeted interventions.

### Implications

Mental health professionals are instrumental in equipping parents with emotional regulation skills and enhancing their empathy, which are crucial for managing the unique challenges of ASD. Parenting programs incorporating evidence-based techniques, such as problem-solving and emotion-focused strategies, can empower parents to address their child’s emotional and behavioral issues effectively. Additionally, these programs should focus on improving communication and resource management to ensure equitable attention among all children in the family. Clinical research should expand to include more trials evaluating the efficacy of family-based interventions, which can provide valuable insights into improving psychological well-being and teaching effective parenting techniques. Furthermore, community health providers should recognize and address the diverse support needs of parents, offering increased psychological support and flexible job skills training. Educational programs and workshops for family members and the public can bridge understanding gaps and foster collaborative decision-making. Finally, Chinese educational systems must offer more inclusive and specialized education to ensure children with ASD have the necessary resources for academic and social success.

## Conclusion

In conclusion, parents reported reduced quality of life when quitting their jobs and sacrificing their time to provide care for autistic children. Parents also experienced psychological problems (e.g., isolation, loneliness, affiliated stigma, and worries) and insufficient support from others. To address these challenges, some parents may adopt helpful coping and parenting strategies, while others may not. Findings suggest that effective support for individuals with autism extends beyond individual-level interventions, necessitating the integration of family care and robust community support systems. Cultivating autism-friendly communities – where understanding, acceptance, and resources are readily available – is crucial for a more inclusive and supportive environment that addresses cultural stigma, empowers caregivers, and ensures access to appropriate services.

## Data Availability

The datasets generated for this study are available upon request from the corresponding author.

## References

[CR1] Alnazly, E. K., & Abojedi, A. (2019). Psychological distress and perceived burden in caregivers of persons with autism spectrum disorder. *Perspectives in Psychiatric Care*, *55*(3), 501–508. 10.1111/ppc.12356 30729536

[CR2] Attard, N., & Booth, N. (2023). Autism and mainstream education: The parental perspective. *International Journal of Educational Research*, *121*, 102234. 10.1016/j.ijer.2023.102234

[CR3] Braun, V., & Clarke, V. (2006). Using thematic analysis in psychology. *Qualitative Research in Psychology*, *3*(2), 77–101. 10.1191/1478088706qp063oa

[CR4] Brown, K. M., Elliott, S. J., Leatherdale, S. T., & Robertson-Wilson, J. (2015). Searching for rigour in the reporting of mixed methods population health research: A methodological review. *Health Education Research*, *30*(6), 811–839. 10.1093/her/cyv046 26491072

[CR5] Brunt, S., Nevill, R., & Mazurek, M. O. (2024). Associations among autism symptom domains and facets of caregiver strain. *Journal of Autism and Developmental Disorders*, *54*(4), 1507–1516. 10.1007/s10803-022-05885-5 PMC10931534 36637596

[CR6] Buono, N., Sassier, B., Thulesius, H., Hoffman, R., Nabbe, P., Petek, D., & Le Reste, J. Y. (2023). Translation of the working alliance inventory short revised into Italian using a Delphi procedure and a forward-backward translation. *Front Med (Lausanne)*, *10*, 1236273. 10.3389/fmed.2023.1236273 PMC10808585 38274448

[CR7] Centers for Disease Control and Prevention (CDC) (2024). *Accessing Services for Autism Spectrum Disorder*. https://www.cdc.gov/autism/treatment/accessing-services.html

[CR8] Chin, W. C., Chang, H. L., & Chao, K. Y. (2023). Exploring coping strategies of parents of children with autism spectrum disorder in Taiwan: A qualitative study. *The Journal of Nursing Research: Jnr*, *31*(3), e278. 10.1097/jnr.0000000000000553 37097915

[CR9] China Daily (2023). *Shanghai, Beijing and Shenzhen top cities in GDP*. https://www.chinadaily.com.cn/a/202301/31/WS63d8c4dca31057c47ebac197.html

[CR10] Cleary, M., West, S., Kornhaber, R., & Hungerford, C. (2023). Autism, discrimination and masking: Disrupting a recipe for trauma. *Issues in Mental Health Nursing*, *44*(9), 799–808. 10.1080/01612840.2023.2239916 37616302

[CR11] Cloete, L. G., & Obaigwa, E. O. (2019). Lived experiences of caregivers of children with autism spectrum disorder in Kenya. *Afr J Disabil*, *8*, 435. 10.4102/ajod.v8i0.435 PMC6489168 31049306

[CR12] Du, S., & Li, Y. (2023). Unequal parenting in China: A study of socio-cultural and political effects. *The Sociological Review*, *72*(3), 691–713. 10.1177/00380261231198329

[CR13] Feng, X., Li, K., Jiang, Q., Zhang, Y., Gong, Z., Zhi, H., Yu, J., Li, W., & Li, J. (2022). Traditional Chinese medicine intervention for autism spectrum disorders: A protocol for systematic review and network meta-analysis. *Medicine (Baltimore)*, *101*(9), e28957. 10.1097/md.0000000000028957 PMC8896422 35244056

[CR14] Ganjeh, F., Fallahi-Khoshknab, M., Riahi, F., Mohammadi, E., & Zarea, K. (2020). Parental concerns about the future of children with autism: A qualitative study in Iran. *International Journal of Ayurvedic Medicine*, *11*, 739–746. 10.47552/ijam.v11i4.1707

[CR15] Gnanasekaran, S., Choueiri, R., Neumeyer, A., Ajari, O., Shui, A., & Kuhlthau, K. (2016). Impact of employee benefits on families with children with autism spectrum disorders. *Autism*, *20*(5), 616–622. 10.1177/1362361315598891 26341992

[CR16] Goh, J. X., Aishworiya, R., Ho, R. C. M., Wang, W., & He, H. G. (2021). A qualitative study exploring experiences and support needs of parents of children with autism spectrum disorder in Singapore. *Journal of Clinical Nursing*, *30*(21–22), 3268–3280. 10.1111/jocn.15836 33969552

[CR17] Guy-Evans, O. (2024). Bronfenbrenner’s Ecological Systems Theory.

[CR20] Han, Q. (2012). An overview of traditional Chinese family ethics. *Quarterly Journal of Chinese Studies*.

[CR18] Han, E., Tan, M. M. J., Crane, L., & Legido-Quigley, H. (2021). A qualitative study of autism services and supports in Singapore: Perspectives of service providers, autistic adults and caregivers. *Autism*, *25*(8), 2279–2290. 10.1177/13623613211016112 34049440

[CR19] Han, J., Hao, Y., Cui, N., Wang, Z., Lyu, P., & Yue, L. (2023). Parenting and parenting resources among Chinese parents with children under three years of age: Rural and urban differences. *BMC Prim Care*, *24*(1), 38. 10.1186/s12875-023-01993-y PMC9890422 36726105

[CR21] Hastings, S. L. (2010). *Encyclopedia of research design*. In. SAGE Publications, Inc. 10.4135/9781412961288

[CR22] Heiman, T. (2021). Parents’ voice: Parents’ emotional and practical coping with a child with special needs. *Psychology*, *12*, 675–691. 10.4236/psych.2021.125042

[CR23] Hennink, M. M., Kaiser, B. N., & Marconi, V. C. (2016). Code saturation versus meaning saturation: How many interviews are enough?? *Qualitative Health Research*, *27*(4), 591–608. 10.1177/1049732316665344 PMC9359070 27670770

[CR24] Horgan, F., Kenny, N., & Flynn, P. (2022). A systematic review of the experiences of autistic young people enrolled in mainstream second-level (post-primary) schools. *Autism*, *27*(2), 526–538. 10.1177/13623613221105089 35757990

[CR25] Johnson, N. L., Krueger, W., Jilek, E., & Haglund, K. (2020). Conversations with health care providers and parents before autism diagnosis: A qualitative study. *Journal of Pediatric Health Care: official Publication of National Association of Pediatric Nurse Associates & Practitioners*, *34*(5), 453–461. 10.1016/j.pedhc.2020.05.002 32674885

[CR26] Khachadourian, V., Mahjani, B., Sandin, S., Kolevzon, A., Buxbaum, J. D., Reichenberg, A., & Janecka, M. (2023). Comorbidities in autism spectrum disorder and their etiologies. *Transl Psychiatry*, *13*(1), 71. 10.1038/s41398-023-02374-w PMC9958310 36841830

[CR27] Kim, J. W., Kim, H. W., Moon, D. S., Lim, Y. S., McDougle, C. J., & Howe, Y. J. (2019). Comparison of services for autism spectrum disorder in Massachusetts with those in Seoul. *Journal of Korean Medical Science*, *34*(43), e288. 10.3346/jkms.2019.34.e288 PMC6838605 31701704

[CR111] Kwok, J. Y. Y., Lee, J. J., Auyeung, M., Chan, M. L., & Chan, H. Y. L. (2020). Letting nature take its course: A qualitative exploration of the illness and adjustment experiences of Hong Kong Chinese people with Parkinson’s disease. *Health Soc Care Community, 28*(6), 2343–2351. 10.1111/hsc.13055 32519426

[CR28] Lao, U., Dai, J., Liang, F., Chen, Q., Cao, W., Pan, Y., Zou, X., & Zhu, H. (2024). Unveiling the perceptions of autism-Related Chinese Language among the autism community and the general public. *Autism in Adulthood*. 10.1089/aut.2024.0001

[CR29] Leaf, J. B., Cihon, J. H., Leaf, R., McEachin, J., Liu, N., Russell, N., Unumb, L., Shapiro, S., & Khosrowshahi, D. (2022). Concerns about ABA-Based intervention: An evaluation and recommendations. *Journal of Autism and Developmental Disorders*, *52*(6), 2838–2853. 10.1007/s10803-021-05137-y PMC9114057 34132968

[CR30] Lee, J. D., Terol, A. K., Yoon, C. D., & Meadan, H. (2024). Parent-to-parent support among parents of children with autism: A review of the literature. *Autism*, *28*(2), 263–275. 10.1177/13623613221146444 36588317

[CR31] Legg, H., & Tickle, A. (2019). UK parents’ experiences of their child receiving a diagnosis of autism spectrum disorder: A systematic review of the qualitative evidence. *Autism*, *23*(8), 1897–1910. 10.1177/1362361319841488 30995082

[CR32] Li, F., Li, Q., Shen, Q., Zhang, X., Leng, H., Liu, Y., & Zheng, X. (2024a). The support needs of parents of children with autism spectrum disorder: A qualitative study based on the nurturing care framework. *Journal of Advanced Nursing*. 10.1111/jan.16681 39696911

[CR33] Li, X., Xu, H., & Zhang, J. (2024b). Attitudes of Chinese public towards the autism community: Evidence from a decade of Weibo data. *Heliyon*, *10*(16), e35113. 10.1016/j.heliyon.2024.e35113 PMC11369461 39229499

[CR35] Lin, M. L., & Faldowski, R. A. (2023). The relationship of parent support and child emotional regulation to school readiness. *International Journal of Environmental Research and Public Health*, *20*(6). 10.3390/ijerph20064867 PMC10049506 36981776

[CR34] Lin, C. R., Tsai, Y. F., & Chang, H. L. (2008). Coping mechanisms of parents of children recently diagnosed with autism in Taiwan: A qualitative study. *Journal of Clinical Nursing*, *17*(20), 2733–2740. 10.1111/j.1365-2702.2008.02456.x 18808642

[CR36] Lincoln, Y. S., & Guba, E. G. (1985). *Naturalistic inquiry*. sage.

[CR39] Liu, Y. (2023). Concerns towards mainstreaming education for students with disabilities in China. *SHS Web of Conferences*, *180*. 10.1051/shsconf/202318002004

[CR38] Liu, X. Y., & To, S. M. (2021). Personal growth experience among parents of children with autism participating in intervention. *Journal of Autism and Developmental Disorders*, *51*(6), 1883–1893. 10.1007/s10803-020-04681-3 32875454

[CR37] Liu, L., & Wang, M. (2018). Parental harsh discipline and adolescent problem behavior in China: Perceived normativeness as a moderator. *Child Abuse & Neglect*, *86*, 1–9. 10.1016/j.chiabu.2018.09.009 30248492

[CR40] Lord, C., & Bishop, S. L. (2015). Recent advances in autism research as reflected in DSM-5 criteria for autism spectrum disorder. *Annu Rev Clin Psychol*, *11*, 53–70. 10.1146/annurev-clinpsy-032814-112745 25581244

[CR41] Lord, C., Elsabbagh, M., Baird, G., & Veenstra-Vanderweele, J. (2018). Autism spectrum disorder. *The Lancet*, *392*(10146), 508–520. 10.1016/S0140-6736(18)31129-2 PMC7398158 30078460

[CR42] Maksimović, S., Marisavljević, M., Stanojević, N., Ćirović, M., Punišić, S., Adamović, T., Đorđević, J., Krgović, I., & Subotić, M. (2023). Importance of early intervention in reducing autistic symptoms and Speech-Language deficits in children with autism spectrum disorder. *Children (Basel)*, *10*(1). 10.3390/children10010122 PMC9857540 36670672

[CR43] Marsack-Topolewski, C. N., Samuel, P. S., & Tarraf, W. (2021). Empirical evaluation of the association between daily living skills of adults with autism and parental caregiver burden. *PLoS One*, *16*(1), e0244844. 10.1371/journal.pone.0244844 PMC7785247 33400706

[CR44] Miranda, A., Mira, A., Berenguer, C., Rosello, B., & Baixauli, I. (2019). Parenting stress in mothers of children with autism without intellectual disability. Mediation of behavioral problems and coping strategies. *Frontiers in Psychology*, *10*, 464. 10.3389/fpsyg.2019.00464 PMC6418028 30906274

[CR45] Ng, C. S. M., & Ng, S. S. L. (2022). A qualitative study on the experience of stigma for Chinese parents of children with autism spectrum disorder. *Scientific Reports*, *12*(1), 19550. 10.1038/s41598-022-23978-0 PMC9666461 36379973

[CR46] Ou, J. J., Shi, L. J., Xun, G. L., Chen, C., Wu, R. R., Luo, X. R., Zhang, F. Y., & Zhao, J. P. (2015). Employment and financial burden of families with preschool children diagnosed with autism spectrum disorders in urban China: Results from a descriptive study. *Bmc Psychiatry*, *15*, 3. 10.1186/s12888-015-0382-4 PMC4307683 25608486

[CR47] Ozdemir, N. K., & Koç, M. (2023). Career adaptability of parents of children with autism spectrum disorder. *Current Psychology*, *42*(17), 14320–14333. 10.1007/s12144-021-02649-9 PMC8791681 35103038

[CR48] Palinkas, L. A., Horwitz, S. M., Green, C. A., Wisdom, J. P., Duan, N., & Hoagwood, K. (2015). Purposeful sampling for qualitative data collection and analysis in mixed method implementation research. *Administration and Policy in Mental Health*, *42*(5), 533–544. 10.1007/s10488-013-0528-y PMC4012002 24193818

[CR49] Rivers, J., & Stoneman, Z. (2008). Child temperaments, differential parenting, and the sibling relationships of children with autism spectrum disorder. *Journal of Autism and Developmental Disorders*, *38*, 1740–1750. 10.1007/s10803-008-0560-z 18369718

[CR50] Salleh, N. S., Abdullah, K. L., Yoong, T. L., Jayanath, S., & Husain, M. (2020). Parents’ experiences of affiliate stigma when caring for a child with autism spectrum disorder (ASD): A Meta-Synthesis of qualitative studies. *Journal of Pediatric Nursing*, *55*, 174–183. 10.1016/j.pedn.2020.09.002 32957021

[CR51] Schnabel, A., Youssef, G. J., Hallford, D. J., Hartley, E. J., McGillivray, J. A., Stewart, M., Forbes, D., & Austin, D. W. (2020). Psychopathology in parents of children with autism spectrum disorder: A systematic review and meta-analysis of prevalence. *Autism*, *24*(1), 26–40. 10.1177/1362361319844636 31070044

[CR52] Smith-Young, J., Pike, A., Swab, M., & Chafe, R. (2024). Parents’ and guardians’ experiences of barriers and facilitators in accessing autism spectrum disorder diagnostic services for their children: A qualitative systematic review. *JBI Evid Synth*. 10.11124/jbies-23-00332 39474706

[CR53] Turnock, A., Langley, K., & Jones, C. R. G. (2022). Understanding stigma in autism: A narrative review and theoretical model. *Autism Adulthood*, *4*(1), 76–91. 10.1089/aut.2021.0005 PMC8992913 36605561

[CR54] Uddin, M. J., & Ashrafun, L. (2023). Parenting in context: Parents’ experiences of caring for a child with autism in Bangladesh. *International Journal of Anthropology and Ethnology*, *7*(1), 10. 10.1186/s41257-023-00089-w

[CR55] Uysal, G., Karakul, A., & Düzkaya, D. S. (2024). Emotions and difficulties experienced by parents of children with autism: A qualitative study. *Journal of Evaluation in Clinical Practice*. 10.1111/jep.14207 39440802

[CR56] Westby, C., Chen, K. M., Cheng, L., Jithavech, P., & Maroonroge, S. (2024). Autism in Taiwan and Thailand: Influences of culture. *Neuropsychiatric Disease and Treatment*, *20*, 1523–1538. 10.2147/ndt.S462864 PMC11303671 39113831

[CR57] Wilson, C. E., Gillan, N., Spain, D., Robertson, D., Roberts, G., Murphy, C. M., Maltezos, S., Zinkstok, J., Johnston, K., Dardani, C., Ohlsen, C., Deeley, P. Q., Craig, M., Mendez, M. A., Happé, F., & Murphy, D. G. (2013). Comparison of ICD-10R, DSM-IV-TR and DSM-5 in an adult autism spectrum disorder diagnostic clinic. *Journal of Autism and Developmental Disorders*, *43*(11), 2515–2525. 10.1007/s10803-013-1799-6 23504376

[CR58] Xue, W. (2024). China special education for disabled children: Problems and countermeasures. *Transactions on Social Science Education and Humanities Research*, *5*, 182–188. 10.62051/zea9h974

[CR59] Yan, T., Hou, Y., & Liang, L. (2023). Family socioeconomic status and parental involvement in Chinese parents of children with autism spectrum disorder: A moderated mediation model. *Healthcare (Basel)*, *11*(9). 10.3390/healthcare11091281 PMC10177892 37174823

[CR60] Yeh, T. C., Chen, M. H., Bai, Y. M., Tsai, S. J., Hsu, J. W., Huang, K. L., Su, T. P., Chen, T. J., & Liang, C. S. (2023). Longitudinal follow-up of subsequent psychiatric comorbidities among children and adolescents with autism spectrum disorder. *Journal of Affective Disorders*, *331*, 245–250. 10.1016/j.jad.2023.03.042 36965622

[CR61] Yim, E. P. (2022). Effects of Asian cultural values on parenting style and young children’s perceived competence: A cross-sectional study. *Frontiers in Psychology*, *13*, 905093. 10.3389/fpsyg.2022.905093 PMC9618636 36324781

[CR62] Yu, L., Stronach, S., & Harrison, A. J. (2020). Public knowledge and stigma of autism spectrum disorder: Comparing China with the united States. *Autism*, *24*(6), 1531–1545. 10.1177/1362361319900839 32338539

[CR63] Zeidan, J., Fombonne, E., Scorah, J., Ibrahim, A., Durkin, M. S., Saxena, S., Yusuf, A., Shih, A., & Elsabbagh, M. (2022). Global prevalence of autism: A systematic review update. *Autism Research*, *15*(5), 778–790. 10.1002/aur.2696 PMC9310578 35238171

[CR64] Zhang, D., Mou, J., Cheng, J. Q., & Griffiths, S. M. (2011). Public health services in Shenzhen: A case study. *Public Health*, *125*(1), 15–19. 10.1016/j.puhe.2010.10.007 PMC7118794 21256365

[CR65] Zhao, Y., Fan, H., Luo, Y., Zhang, R., & Zheng, X. (2024). Gender inequalities in employment of parents caring for children with autism spectrum disorder in China: Cross-Sectional study. *JMIR Pediatr Parent*, *7*, e59696. 10.2196/59696 PMC11683506 39801142

